# 2-[3-((*Z*)-2-{4-[Bis(2-chloro­eth­yl)amino]­phen­yl}ethen­yl)-5,5-dimethyl­cyclo­hex-2-en-1-yl­idene]propane­dinitrile

**DOI:** 10.1107/S1600536810051068

**Published:** 2010-12-15

**Authors:** Li Liu, Ying Shao, Jie-Ping Shi, Hong-Wen Hu, Guo-Yuan Lu

**Affiliations:** a State Key Laboratory of Coordination Chemistry, School of Chemistry and Chemical Engineering, Nanjing University, Nanjing 210093 Jiangsu, People’s Republic of China; b Key Laboratory of Fine Chemical Engineering, Changzhou University, Changzhou 213164 Jiangsu, People’s Republic of China

## Abstract

The highly conjugated title compound, C_23_H_25_Cl_2_N_3_, is nearly planar (the mean deviation from the plane being 0.049 Å), except for the –C(CH_3_)_2_ group on the cyclo­hexene ring and the two CH_2_Cl groups. The cyclo­hexene ring has an envelope configuration. In the crystal, the packing is stabilized by C—H⋯Cl inter­actions and C—H⋯π inter­actions involving the benzene ring.

## Related literature

The title compound was prepared by the Knoevenagel reaction, see: Bai *et al.* (2006 ▶); Samyn *et al.* (2001 ▶). It is an inter­mediate for the preparation of non-linear optical materials, see: Kwon *et al.* (2006 ▶); Shu *et al.* (1998 ▶); Chun *et al.* (2001 ▶); Zheng *et al.* (2000 ▶). For a related structure, see Kolev *et al.* (2005 ▶).
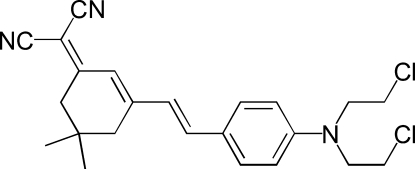


## Experimental

### 

#### Crystal data

C_23_H_25_Cl_2_N_3_
*M* *_r_* = 414.36
Triclinic, 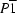

*a* = 9.106 (7) Å
*b* = 10.819 (9) Å
*c* = 13.325 (4) Å
α = 70.052 (6)°
β = 70.02 (1)°
γ = 65.11 (1)°
*V* = 1088.8 (13) Å^3^
*Z* = 2
Mo *K*α radiation
μ = 0.31 mm^−1^
*T* = 295 K
0.25 × 0.20 × 0.15 mm

#### Data collection

Bruker SMART CCD area-detector diffractometer
Absorption correction: multi-scan (*SADABS*; Bruker, 2000 ▶) *T*_min_ = 0.926, *T*_max_ = 0.955
5376 measured reflections
3695 independent reflections
3130 reflections with *I* > 2σ(*I*)
*R*_int_ = 0.108

#### Refinement

*R*[*F* ^2^ > 2σ(*F* ^2^)] = 0.052
*wR*(*F* ^2^) = 0.149
*S* = 1.08
3695 reflections
253 parameters
H-atom parameters constrained
Δρ_max_ = 0.40 e Å^−3^
Δρ_min_ = −0.45 e Å^−3^

### 

Data collection: *SMART* (Bruker, 2000 ▶); cell refinement: *SAINT* (Bruker, 2000 ▶); data reduction: *SAINT*; program(s) used to solve structure: *SHELXS97* (Sheldrick, 2008 ▶); program(s) used to refine structure: *SHELXL97* (Sheldrick, 2008 ▶); molecular graphics: *SHELXTL* (Sheldrick, 2008 ▶); software used to prepare material for publication: *SHELXTL*.

## Supplementary Material

Crystal structure: contains datablocks I, global. DOI: 10.1107/S1600536810051068/fl2317sup1.cif

Structure factors: contains datablocks I. DOI: 10.1107/S1600536810051068/fl2317Isup2.hkl

Additional supplementary materials:  crystallographic information; 3D view; checkCIF report

## Figures and Tables

**Table 1:** Hydrogen-bond geometry (Å, °) *Cg*1 is the centroid of the C11–C16 ring.

*D*—H⋯*A*	*D*—H	H⋯*A*	*D*⋯*A*	*D*—H⋯*A*
C18—H18*B*⋯Cl1B^i^	0.97	2.91	3.822 (2)	158
C4—H4*A*⋯*Cg*1^ii^	0.97	2.55	3.459 (2)	156

Symmetry codes: (i) 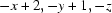; (ii) 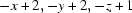.

**Fig. 1.**
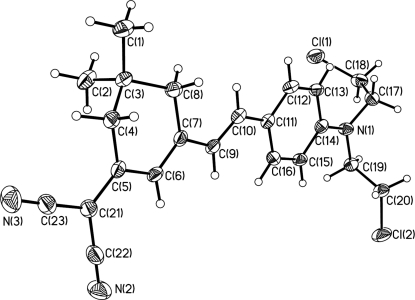
View of the title compound, showing the labeling of the non-H atoms and 50% probability ellipsoids.

**Fig. 2.**
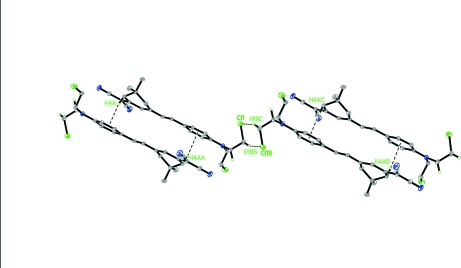
Tetrameric subunits linked by C—H···Cl and C—H···π interactions in the title compound. H atoms not involved in short contacts have been omitted for clarity.

## supplementary crystallographic information

### Comment

The title compound, (I), was prepared by the Knoevenagel reaction (Bai *et al.*, 2006; Samyn *et al.*, 2001). With a donor-π-acceptor (D-π-A) structure, it is one of the important intermediates used in nonlinear optical materials (Kwon *et al.*, 2006; Shu *et al.*, 1998; Chun *et al.*, 2001; Zheng *et al.*, 2000). We now report the structure (I) (Fig. 1). The C—N1 bond length is shorter than a normal single C—N bond (1.47–1.50 Å) and longer than a double C=N bond distance (1.34–1.38 Å) which is due to the *p*-π conjugation in the phenyl amine group. Because of the extended conjugation, almost all atoms in the molecule are roughly coplanar, except for the C(CH_3_)_2_ and CH_2_Cl groups. The cyclohexene ring adopts an envelope configuration due to its ring tension, with atom C3 deviating by 0.635 (2) Å from the mean plane through the remaining atoms. The CH_2_Cl groups are on opposite sides of the plane, the N—C—C—Cl torsion angles are 64.5 (2)° for Cl1—C18—C17—N1 and 173.0 (1)° for Cl2—C20—C19—N1. The structure of a related compound having a diphenyl group instead of the chloroethyl moiety has been reported (Kolev *et al.*, 2005). In the crystal structure of (I), no hydrogen bonding is found. The crystal packing is stabilized by C—H···Cl interactions and C—H···π interactions involving the benzene ring (Table 1, Fig. 2). For the C—H···π interactions, the relevant distances and angles are: C···*Cg*^[i]^ = 3.459 (4) Å, H···*Cg*^[i]^ =2.548 (2)Å and C—H···*Cg*^[i]^= 156 (1)° [symmetry code: (i) 2 - *x*, 2 - *y*, 1 - *z*].

### Experimental

To a solution of 4-(bis(2-chloroethyl)amino)benzaldehyde (1.0 g, 4.1 mmol) in 10 ml anhydrous DMF, 2-(3,5,5-trimethylcyclohex-2-enylidene)malononitrile (0.93 g, 5.0 mmol), 0.5 ml acetic acid, 1 ml piperidine were added, respectively. The reaction mixture was stirred for 2 days at room temperature. Then, the mixture was poured into 50 ml of water and filtered. The resulting solid was purified by column chromatography (petroleum ether/acetic ester, 5:1). Red product 0.24 g was obtained. Yield: 14.2%. Single crystals suitable for X-ray diffraction were obtained by slow evaporation of the eluate.

### Refinement

All the H atoms were placed in geometrically idealized positions and constrained to ride on their parent atoms, with C—H distances of 0.93–0.98 Å, and with *U*_iso_(H) = 1.2*U*_eq_(C).

### Crystal data

**Table:**

C_23_H_25_Cl_2_N_3_	*Z* = 2
*M**_r_* = 414.36	*F*(000) = 436
Triclinic, *P*1	*D*_x_ = 1.264 Mg m^−^^3^
Hall symbol: -P 1	Melting point: 462(2) K
*a* = 9.106 (7) Å	Mo *K*α radiation, λ = 0.71073 Å
*b* = 10.819 (9) Å	Cell parameters from 3172 reflections
*c* = 13.325 (4) Å	θ = 2.4–28.3°
α = 70.052 (6)°	µ = 0.31 mm^−^^1^
β = 70.02 (1)°	*T* = 295 K
γ = 65.11 (1)°	Block, red
*V* = 1088.8 (13) Å^3^	0.25 × 0.20 × 0.15 mm

### Data collection

**Table:**

Bruker SMART CCD area-detector diffractometer	3695 independent reflections
Radiation source: fine-focus sealed tube	3130 reflections with *I* > 2σ(*I*)
graphite	*R*_int_ = 0.108
phi and ω scans	θ_max_ = 25.0°, θ_min_ = 1.7°
Absorption correction: multi-scan (*SADABS*; Bruker, 2000)	*h* = −10→9
*T*_min_ = 0.926, *T*_max_ = 0.955	*k* = −12→12
5376 measured reflections	*l* = −15→15

### Refinement

**Table:**

Refinement on *F*^2^	Primary atom site location: structure-invariant direct methods
Least-squares matrix: full	Secondary atom site location: difference Fourier map
*R*[*F*^2^ > 2σ(*F*^2^)] = 0.052	Hydrogen site location: inferred from neighbouring sites
*wR*(*F*^2^) = 0.149	H-atom parameters constrained
*S* = 1.08	*w* = 1/[σ^2^(*F*_o_^2^) + (0.0923*P*)^2^ + 0.0577*P*] where *P* = (*F*_o_^2^ + 2*F*_c_^2^)/3
3695 reflections	(Δ/σ)_max_ < 0.001
253 parameters	Δρ_max_ = 0.40 e Å^−^^3^
0 restraints	Δρ_min_ = −0.45 e Å^−^^3^

### Special details

**Table:**

Geometry. All e.s.d.'s (except the e.s.d. in the dihedral angle between two l.s. planes) are estimated using the full covariance matrix. The cell e.s.d.'s are taken into account individually in the estimation of e.s.d.'s in distances, angles and torsion angles; correlations between e.s.d.'s in cell parameters are only used when they are defined by crystal symmetry. An approximate (isotropic) treatment of cell e.s.d.'s is used for estimating e.s.d.'s involving l.s. planes.
Refinement. Refinement of *F*^2^ against ALL reflections. The weighted *R*-factor *wR* and goodness of fit *S* are based on *F*^2^, conventional *R*-factors *R* are based on *F*, with *F* set to zero for negative *F*^2^. The threshold expression of *F*^2^ > σ(*F*^2^) is used only for calculating *R*-factors(gt) *etc*. and is not relevant to the choice of reflections for refinement. *R*-factors based on *F*^2^ are statistically about twice as large as those based on *F*, and *R*- factors based on ALL data will be even larger.

### Fractional atomic coordinates and isotropic or equivalent isotropic displacement parameters (Å^2^)

**Table:**

	*x*	*y*	*z*	*U*_iso_*/*U*_eq_	
Cl1	0.94296 (7)	0.45978 (6)	0.17603 (5)	0.0391 (2)	
Cl2	1.69804 (7)	0.19448 (6)	0.30464 (5)	0.0455 (2)	
N1	1.2573 (2)	0.45672 (17)	0.23659 (13)	0.0238 (4)	
C6	0.8407 (2)	0.95499 (19)	0.75122 (15)	0.0215 (4)	
H6A	0.9244	0.8771	0.7786	0.026*	
C7	0.8134 (2)	0.9638 (2)	0.65538 (15)	0.0208 (4)	
C14	1.1692 (2)	0.55189 (19)	0.30229 (15)	0.0216 (4)	
C5	0.7456 (2)	1.0609 (2)	0.81253 (15)	0.0210 (4)	
C12	0.9649 (2)	0.7739 (2)	0.33495 (16)	0.0231 (4)	
H12A	0.8866	0.8586	0.3097	0.028*	
C10	0.9014 (2)	0.8540 (2)	0.50045 (16)	0.0224 (4)	
H10A	0.8225	0.9330	0.4692	0.027*	
C16	1.1131 (2)	0.6197 (2)	0.47076 (16)	0.0229 (4)	
H16A	1.1352	0.5974	0.5392	0.027*	
C11	0.9938 (2)	0.7485 (2)	0.43735 (15)	0.0215 (4)	
C9	0.9138 (2)	0.8536 (2)	0.59796 (16)	0.0222 (4)	
H9A	0.9928	0.7767	0.6308	0.027*	
C13	1.0470 (2)	0.6794 (2)	0.26958 (15)	0.0231 (4)	
H13	1.0211	0.7003	0.2026	0.028*	
C21	0.7696 (2)	1.0458 (2)	0.91198 (16)	0.0240 (4)	
C23	0.6733 (3)	1.1514 (2)	0.97320 (16)	0.0281 (5)	
C15	1.1987 (2)	0.5248 (2)	0.40534 (16)	0.0229 (4)	
H15A	1.2781	0.4407	0.4302	0.028*	
C4	0.6212 (2)	1.1924 (2)	0.76371 (16)	0.0238 (4)	
H4A	0.6762	1.2596	0.7189	0.029*	
H4B	0.5353	1.2329	0.8223	0.029*	
C19	1.3873 (2)	0.3277 (2)	0.27018 (16)	0.0241 (4)	
H19A	1.4040	0.2599	0.2312	0.029*	
H19B	1.3531	0.2894	0.3481	0.029*	
N3	0.5935 (3)	1.2370 (2)	1.02102 (15)	0.0399 (5)	
C8	0.6800 (2)	1.0893 (2)	0.60875 (16)	0.0252 (4)	
H8A	0.6307	1.0594	0.5713	0.030*	
H8B	0.7312	1.1548	0.5544	0.030*	
C3	0.5410 (2)	1.1659 (2)	0.69367 (16)	0.0236 (4)	
C17	1.2492 (3)	0.4899 (2)	0.12292 (16)	0.0261 (5)	
H17A	1.3614	0.4675	0.0772	0.031*	
H17B	1.1921	0.5898	0.1000	0.031*	
C18	1.1606 (3)	0.4119 (2)	0.10506 (18)	0.0323 (5)	
H18A	1.2152	0.3121	0.1304	0.039*	
H18B	1.1691	0.4308	0.0271	0.039*	
N2	0.9835 (3)	0.8257 (2)	0.99930 (16)	0.0436 (5)	
C22	0.8897 (3)	0.9239 (2)	0.96049 (16)	0.0290 (5)	
C20	1.5502 (2)	0.3513 (2)	0.24686 (18)	0.0286 (5)	
H20A	1.5316	0.4267	0.2785	0.034*	
H20B	1.5926	0.3778	0.1682	0.034*	
C1	0.4348 (3)	1.3060 (2)	0.63482 (18)	0.0342 (5)	
H1A	0.5036	1.3608	0.5891	0.051*	
H1B	0.3498	1.3553	0.6880	0.051*	
H1C	0.3839	1.2901	0.5902	0.051*	
C2	0.4306 (3)	1.0779 (2)	0.76748 (19)	0.0338 (5)	
H2A	0.3449	1.1278	0.8200	0.051*	
H2B	0.4975	0.9902	0.8053	0.051*	
H2C	0.3807	1.0607	0.7231	0.051*	

### Atomic displacement parameters (Å^2^)

**Table:**

	*U*^11^	*U*^22^	*U*^33^	*U*^12^	*U*^13^	*U*^23^
Cl1	0.0324 (3)	0.0429 (4)	0.0442 (4)	−0.0126 (3)	−0.0161 (3)	−0.0062 (3)
Cl2	0.0276 (3)	0.0419 (4)	0.0561 (4)	−0.0014 (3)	−0.0205 (3)	−0.0012 (3)
N1	0.0230 (9)	0.0203 (8)	0.0260 (9)	−0.0007 (7)	−0.0093 (7)	−0.0077 (7)
C6	0.0148 (9)	0.0188 (9)	0.0262 (10)	−0.0013 (8)	−0.0050 (8)	−0.0051 (8)
C7	0.0155 (9)	0.0237 (10)	0.0215 (10)	−0.0078 (8)	−0.0010 (8)	−0.0052 (8)
C14	0.0192 (10)	0.0201 (10)	0.0264 (11)	−0.0076 (8)	−0.0039 (8)	−0.0066 (8)
C5	0.0155 (9)	0.0218 (10)	0.0230 (10)	−0.0066 (8)	−0.0007 (8)	−0.0054 (8)
C12	0.0176 (9)	0.0205 (10)	0.0306 (11)	−0.0027 (8)	−0.0090 (8)	−0.0068 (8)
C10	0.0168 (9)	0.0211 (10)	0.0279 (11)	−0.0054 (8)	−0.0036 (8)	−0.0069 (8)
C16	0.0228 (10)	0.0234 (10)	0.0207 (10)	−0.0066 (8)	−0.0051 (8)	−0.0048 (8)
C11	0.0166 (9)	0.0226 (10)	0.0245 (10)	−0.0068 (8)	−0.0030 (8)	−0.0062 (8)
C9	0.0144 (9)	0.0228 (10)	0.0251 (10)	−0.0034 (8)	−0.0035 (8)	−0.0055 (8)
C13	0.0203 (10)	0.0258 (10)	0.0229 (10)	−0.0040 (8)	−0.0089 (8)	−0.0066 (8)
C21	0.0236 (10)	0.0220 (10)	0.0227 (10)	−0.0050 (8)	−0.0049 (8)	−0.0049 (8)
C23	0.0296 (11)	0.0281 (11)	0.0225 (10)	−0.0079 (9)	−0.0051 (9)	−0.0048 (9)
C15	0.0199 (10)	0.0184 (10)	0.0255 (10)	−0.0037 (8)	−0.0084 (8)	0.0002 (8)
C4	0.0214 (10)	0.0216 (10)	0.0242 (10)	−0.0032 (8)	−0.0037 (8)	−0.0072 (8)
C19	0.0236 (10)	0.0188 (10)	0.0275 (10)	−0.0040 (8)	−0.0065 (8)	−0.0064 (8)
N3	0.0463 (12)	0.0337 (11)	0.0319 (10)	−0.0050 (9)	−0.0035 (9)	−0.0153 (9)
C8	0.0226 (10)	0.0261 (10)	0.0257 (10)	−0.0040 (9)	−0.0081 (8)	−0.0076 (8)
C3	0.0169 (9)	0.0238 (10)	0.0255 (10)	−0.0028 (8)	−0.0058 (8)	−0.0047 (8)
C17	0.0270 (10)	0.0221 (10)	0.0248 (10)	−0.0039 (9)	−0.0059 (9)	−0.0060 (8)
C18	0.0338 (12)	0.0320 (11)	0.0313 (11)	−0.0044 (10)	−0.0137 (10)	−0.0105 (9)
N2	0.0468 (12)	0.0374 (11)	0.0357 (11)	0.0032 (10)	−0.0214 (10)	−0.0062 (9)
C22	0.0338 (12)	0.0310 (12)	0.0206 (10)	−0.0080 (10)	−0.0058 (9)	−0.0085 (9)
C20	0.0230 (10)	0.0258 (11)	0.0326 (11)	−0.0032 (9)	−0.0097 (9)	−0.0048 (9)
C1	0.0275 (11)	0.0303 (12)	0.0360 (12)	0.0020 (9)	−0.0116 (10)	−0.0081 (10)
C2	0.0190 (10)	0.0352 (12)	0.0431 (13)	−0.0079 (9)	−0.0035 (9)	−0.0097 (10)

### Geometric parameters (Å, °)

**Table:**

Cl1—C18	1.811 (3)	C23—N3	1.146 (3)
Cl2—C20	1.783 (2)	C15—H15A	0.9300
N1—C14	1.382 (3)	C4—C3	1.520 (3)
N1—C19	1.448 (2)	C4—H4A	0.9700
N1—C17	1.453 (2)	C4—H4B	0.9700
C6—C7	1.347 (3)	C19—C20	1.520 (3)
C6—C5	1.432 (3)	C19—H19A	0.9700
C6—H6A	0.9300	C19—H19B	0.9700
C7—C9	1.438 (3)	C8—C3	1.532 (3)
C7—C8	1.503 (3)	C8—H8A	0.9700
C14—C15	1.398 (3)	C8—H8B	0.9700
C14—C13	1.400 (3)	C3—C1	1.523 (3)
C5—C21	1.360 (3)	C3—C2	1.542 (3)
C5—C4	1.499 (3)	C17—C18	1.504 (3)
C12—C13	1.372 (3)	C17—H17A	0.9700
C12—C11	1.391 (3)	C17—H17B	0.9700
C12—H12A	0.9300	C18—H18A	0.9700
C10—C9	1.340 (3)	C18—H18B	0.9700
C10—C11	1.442 (3)	N2—C22	1.135 (3)
C10—H10A	0.9300	C20—H20A	0.9700
C16—C15	1.376 (3)	C20—H20B	0.9700
C16—C11	1.398 (3)	C1—H1A	0.9600
C16—H16A	0.9300	C1—H1B	0.9600
C9—H9A	0.9300	C1—H1C	0.9600
C13—H13	0.9300	C2—H2A	0.9600
C21—C22	1.428 (3)	C2—H2B	0.9600
C21—C23	1.430 (3)	C2—H2C	0.9600
			
C14—N1—C19	121.33 (16)	C20—C19—H19A	109.3
C14—N1—C17	122.58 (16)	N1—C19—H19B	109.3
C19—N1—C17	115.10 (15)	C20—C19—H19B	109.3
C7—C6—C5	122.55 (17)	H19A—C19—H19B	108.0
C7—C6—H6A	118.7	C7—C8—C3	114.66 (16)
C5—C6—H6A	118.7	C7—C8—H8A	108.6
C6—C7—C9	119.65 (17)	C3—C8—H8A	108.6
C6—C7—C8	119.76 (17)	C7—C8—H8B	108.6
C9—C7—C8	120.58 (16)	C3—C8—H8B	108.6
N1—C14—C15	121.12 (17)	H8A—C8—H8B	107.6
N1—C14—C13	122.14 (17)	C4—C3—C1	108.99 (17)
C15—C14—C13	116.74 (17)	C4—C3—C8	108.14 (16)
C21—C5—C6	121.30 (18)	C1—C3—C8	109.44 (16)
C21—C5—C4	119.90 (17)	C4—C3—C2	109.59 (17)
C6—C5—C4	118.78 (16)	C1—C3—C2	109.60 (17)
C13—C12—C11	122.83 (18)	C8—C3—C2	111.03 (17)
C13—C12—H12A	118.6	N1—C17—C18	112.94 (17)
C11—C12—H12A	118.6	N1—C17—H17A	109.0
C9—C10—C11	128.83 (18)	C18—C17—H17A	109.0
C9—C10—H10A	115.6	N1—C17—H17B	109.0
C11—C10—H10A	115.6	C18—C17—H17B	109.0
C15—C16—C11	121.91 (18)	H17A—C17—H17B	107.8
C15—C16—H16A	119.0	C17—C18—Cl1	112.66 (15)
C11—C16—H16A	119.0	C17—C18—H18A	109.1
C12—C11—C16	115.97 (17)	Cl1—C18—H18A	109.1
C12—C11—C10	118.97 (17)	C17—C18—H18B	109.1
C16—C11—C10	125.06 (17)	Cl1—C18—H18B	109.1
C10—C9—C7	124.63 (18)	H18A—C18—H18B	107.8
C10—C9—H9A	117.7	N2—C22—C21	178.7 (2)
C7—C9—H9A	117.7	C19—C20—Cl2	109.49 (15)
C12—C13—C14	120.96 (17)	C19—C20—H20A	109.8
C12—C13—H13	119.5	Cl2—C20—H20A	109.8
C14—C13—H13	119.5	C19—C20—H20B	109.8
C5—C21—C22	121.90 (18)	Cl2—C20—H20B	109.8
C5—C21—C23	121.02 (18)	H20A—C20—H20B	108.2
C22—C21—C23	117.08 (17)	C3—C1—H1A	109.5
N3—C23—C21	178.5 (2)	C3—C1—H1B	109.5
C16—C15—C14	121.56 (17)	H1A—C1—H1B	109.5
C16—C15—H15A	119.2	C3—C1—H1C	109.5
C14—C15—H15A	119.2	H1A—C1—H1C	109.5
C5—C4—C3	112.19 (16)	H1B—C1—H1C	109.5
C5—C4—H4A	109.2	C3—C2—H2A	109.5
C3—C4—H4A	109.2	C3—C2—H2B	109.5
C5—C4—H4B	109.2	H2A—C2—H2B	109.5
C3—C4—H4B	109.2	C3—C2—H2C	109.5
H4A—C4—H4B	107.9	H2A—C2—H2C	109.5
N1—C19—C20	111.41 (16)	H2B—C2—H2C	109.5
N1—C19—H19A	109.3		
			
C5—C6—C7—C9	179.91 (16)	C6—C5—C21—C23	−179.48 (17)
C5—C6—C7—C8	0.6 (3)	C4—C5—C21—C23	2.4 (3)
C19—N1—C14—C15	−2.1 (3)	C11—C16—C15—C14	−0.8 (3)
C17—N1—C14—C15	−170.09 (17)	N1—C14—C15—C16	179.16 (17)
C19—N1—C14—C13	177.77 (17)	C13—C14—C15—C16	−0.7 (3)
C17—N1—C14—C13	9.8 (3)	C21—C5—C4—C3	−148.39 (18)
C7—C6—C5—C21	176.34 (18)	C6—C5—C4—C3	33.4 (2)
C7—C6—C5—C4	−5.5 (3)	C14—N1—C19—C20	−81.3 (2)
C13—C12—C11—C16	0.1 (3)	C17—N1—C19—C20	87.5 (2)
C13—C12—C11—C10	−179.90 (17)	C6—C7—C8—C3	−24.2 (3)
C15—C16—C11—C12	1.1 (3)	C9—C7—C8—C3	156.51 (17)
C15—C16—C11—C10	−178.87 (18)	C5—C4—C3—C1	−172.37 (16)
C9—C10—C11—C12	−177.25 (19)	C5—C4—C3—C8	−53.5 (2)
C9—C10—C11—C16	2.7 (3)	C5—C4—C3—C2	67.7 (2)
C11—C10—C9—C7	−179.01 (17)	C7—C8—C3—C4	49.7 (2)
C6—C7—C9—C10	−177.52 (18)	C7—C8—C3—C1	168.33 (16)
C8—C7—C9—C10	1.8 (3)	C7—C8—C3—C2	−70.5 (2)
C11—C12—C13—C14	−1.7 (3)	C14—N1—C17—C18	−110.7 (2)
N1—C14—C13—C12	−177.95 (18)	C19—N1—C17—C18	80.6 (2)
C15—C14—C13—C12	1.9 (3)	N1—C17—C18—Cl1	64.6 (2)
C6—C5—C21—C22	−0.2 (3)	N1—C19—C20—Cl2	172.98 (13)
C4—C5—C21—C22	−178.39 (18)		

### Hydrogen-bond geometry (Å, °)

**Table:**

Cg1 is the centroid of the C11–C16 ring.

**Table:**

*D*—H···*A*	*D*—H	H···*A*	*D*···*A*	*D*—H···*A*
C18—H18B···Cl1B^i^	0.97	2.91	3.822 (2)	158
C4—H4A···Cg1^ii^	0.97	2.55	3.459 (2)	156

Symmetry codes: (i) −*x*+2, −*y*+1, −*z*; (ii) −*x*+2, −*y*+2, −*z*+1.
